# Inequalities in adverse birth outcomes and survival in early childhood: birth cohort in South Korea

**DOI:** 10.1093/eurpub/ckac129.654

**Published:** 2022-10-25

**Authors:** M Jo, H Chung

**Affiliations:** 1 Department of Public Health Sciences, Graduate School of Korea University, Seoul, South Korea; 2 BK21FOUR Learning Health Systems, Korea University, Seoul, South Korea; 3 School of Health Policy and Management, Korea University, Seoul, South Korea

## Abstract

**Background:**

Adverse birth outcomes (ABOs) are considered the most common factor of deaths in early childhood. Inequalities in child mortality occur due to interactions between intrinsic and socio-environmental factors related to socioeconomic disadvantage. There are, however, few studies investigating the impact of ABOs on mortality in terms of parental SEP.

**Methods:**

Using the Under-5 Infant Birth-Death Cohort Data in Korea, a pooled retrospective birth cohort of all children born in 2012-2014 was built (N = 1,356,584). We analyzed neonatal, post-neonatal, and childhood mortality by ABOs and with the interaction of parental SEP using the Cox proportional hazard regression model for survival analyses. We further stratified the analysis both by parental SEP and child age. Multiple logistic regression was performed to confirm the social inequalities in ABO itself.

**Results:**

After adjusting for covariates, children born with ABOs presented higher risk of mortality for all periods. For post-neonatal period, lower maternal education showed significant interaction effect with LBW (HR = 0.57; 95% CI = [0.39-0.85]), PTB (HR = 0.53; 95% CI = [0.33-0.86]), LBW & PTB ([HR = 0.67; 95% CI = [0.54-0.83]) while lower paternal education (HR = 0.67; 95% CI = [0.54-0.82]) and maternal unemployment (HR = 0.80; 95% CI = [0.63-0.99]) showed significance for babies with LBW & PTB. However, stratification analyses suggested that the impact of ABOs on mortality was greater for children born to lower parental SEP in neonatal period. Meanwhile apparent social inequalities in ABOs were suggested from regression analyses.

**Conclusions:**

We confirmed social inequalities in the incidence of ABOs as well as mortalities from ABOs. However, the difference in mortality between babies with and without ABOs was greater for advantaged children. Policies to reduce the mortality of children with ABOs as well as those of healthy children among socioeconomically disadvantaged families are required.

**Key messages:**

• Social inequalities in mortality from ABOs were apparent especially in the neonatal period while the incidence of ABOs itself was greater among children from disadvantaged families.

• Disadvantaged children are more likely to die not only from ABOs but also from other socio-environmental determinants, especially in the post-neonatal period than their counterparts.

